# Supplementary material for: Structure-based prediction and characterization of photo-crosslinking in native protein–RNA complexes
Source: Nat Commun. 2024 Mar 13;15:2279. doi: 10.1038/s41467-024-46429-y (PMC10937933; doi:10.1038/s41467-024-46429-y)
Supplement: Supplementary file 1 — Supplementary Information [file 41467_2024_46429_MOESM1_ESM.pdf]

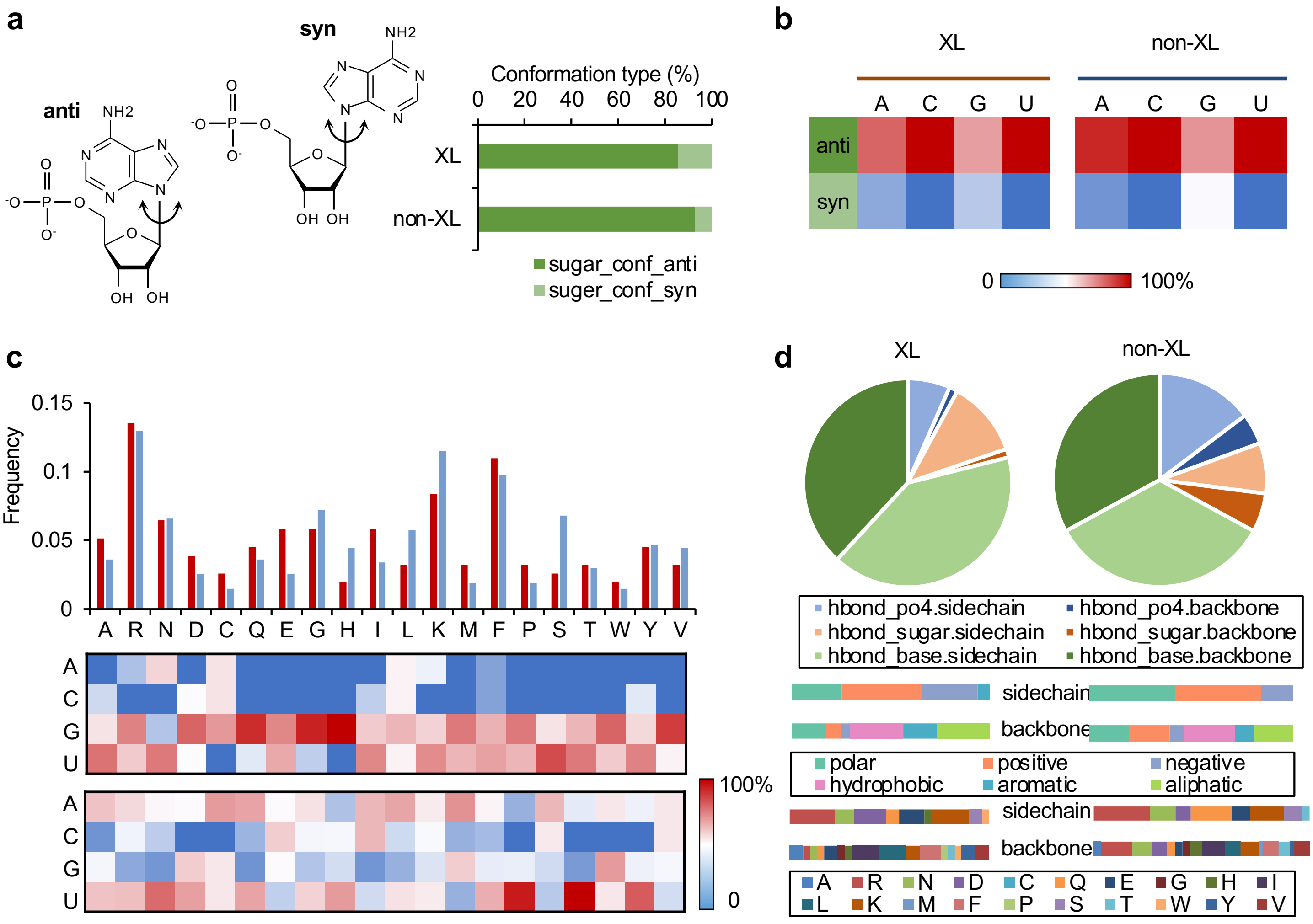

Supplementary Fig. 1: Characterization of crosslinked nucleotides and the associated structural features. **a**, Distribution of sugar conformation types for crosslinked and non-crosslinked nucleotides contacting amino acids in protein-RNA complex structures. The schematics of syn- and anti-conformations are shown on the left. **b**, Similar to (a), but distributions for individual nucleotide bases are shown separately using heatmaps. **c**, Distribution of amino acids contacting with crosslinked and non-crosslinked nucleotides. **d**, Distribution of hydrogen bond types formed between amino acids and crosslinked vs. non-crosslinked nucleotides.

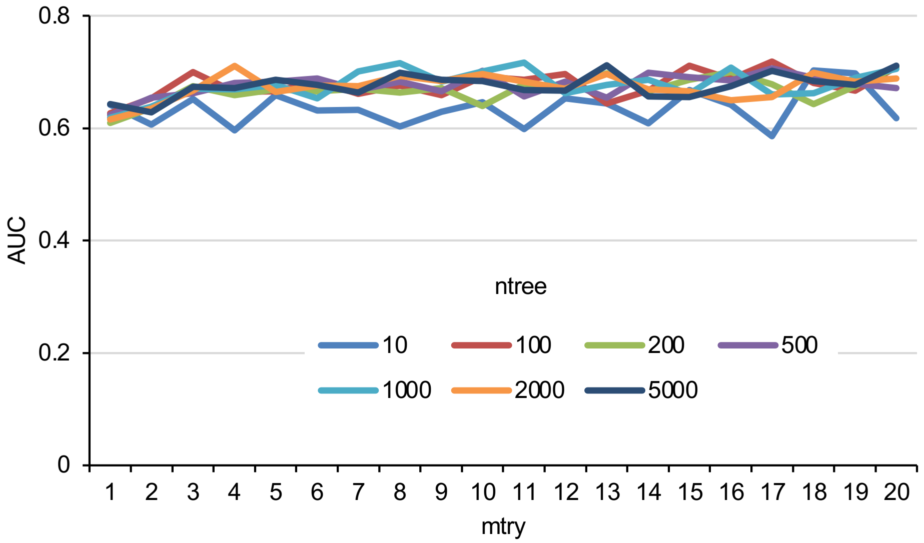

Supplementary Fig. 2: Prediction performance of crosslinked vs. non-crosslinked nucleotides using random forest models trained with different model parameters. A wide range of model parameters including the number of trees (ntree) in the forest and the number of features per tree (mtry) are tested, and the AUC of each model is plotted.

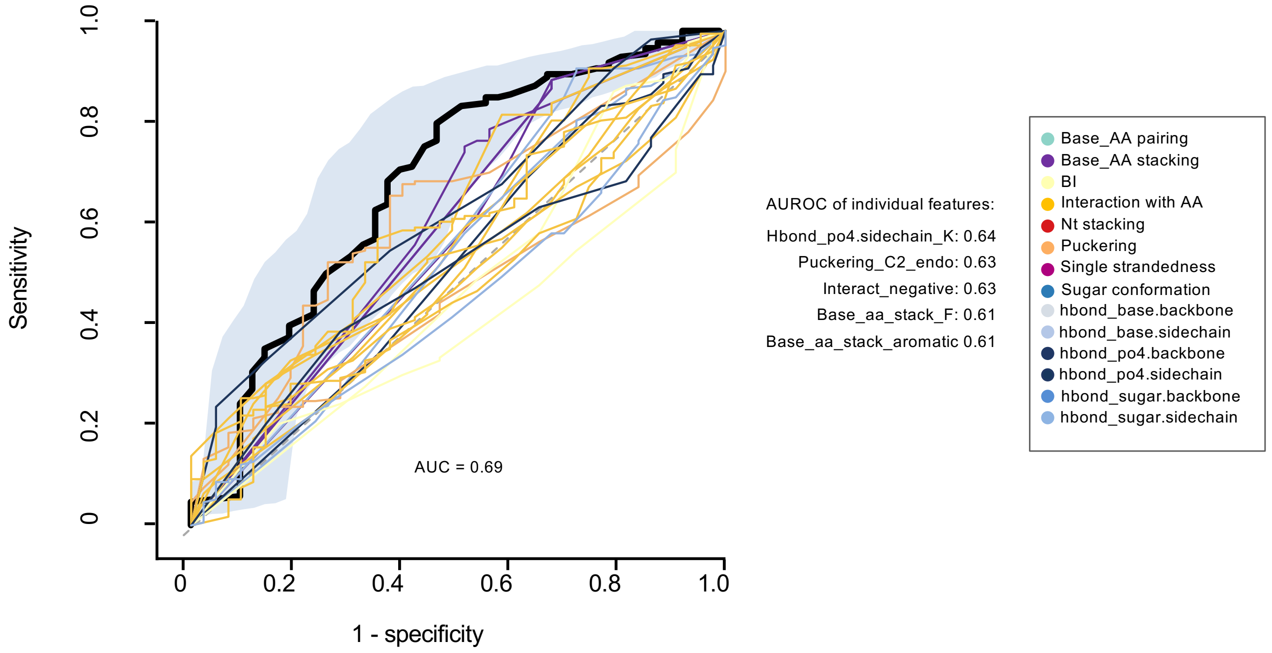

Supplementary Fig. 3: Prediction performance of crosslinked vs. non-crosslinked nucleotides using all features combined or individual features. The ROC of the random forest model using all features is shown by the black curve. The shaded area indicates 95% confidence interval as determined by 2000 models trained with bootstrapped data. The ROCs of individual features are also shown, with color codes indicated on the right. The AUCs of the top individual features are also indicated.

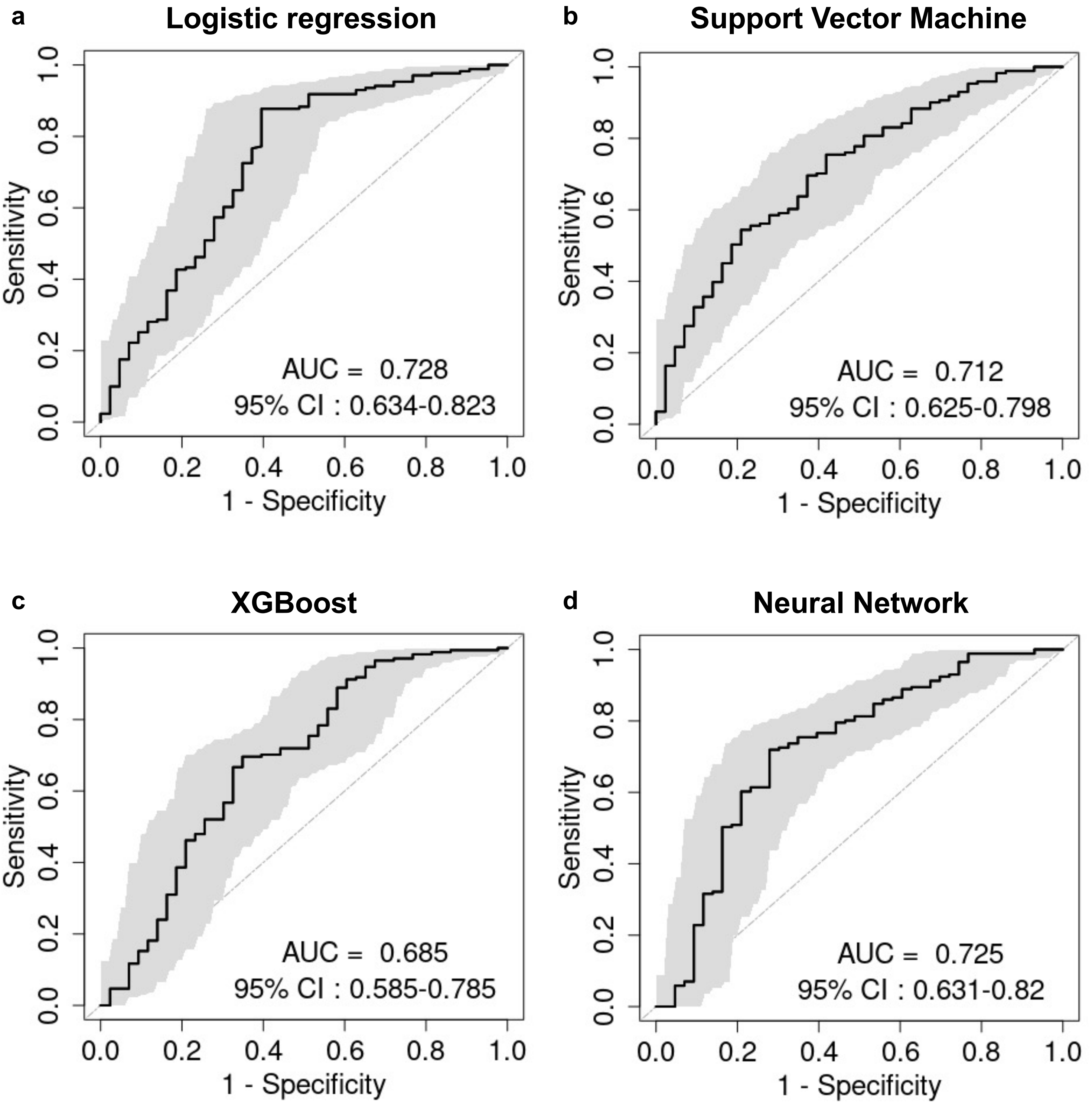

Supplementary Fig. 4: Prediction performance of crosslinked vs. non-crosslinked nucleotides using different classification methods. **a**, Logistic regression. **b**, Support vector machine (SVM). **c**, XGBoost. **d**, Neural network. In each panel, the prediction performance of crosslinked vs. non-crosslinked nucleotides is measured by ROC AUC (black curve). The shaded area indicates 95% confidence interval as determined by 2000 models trained with bootstrapped data.

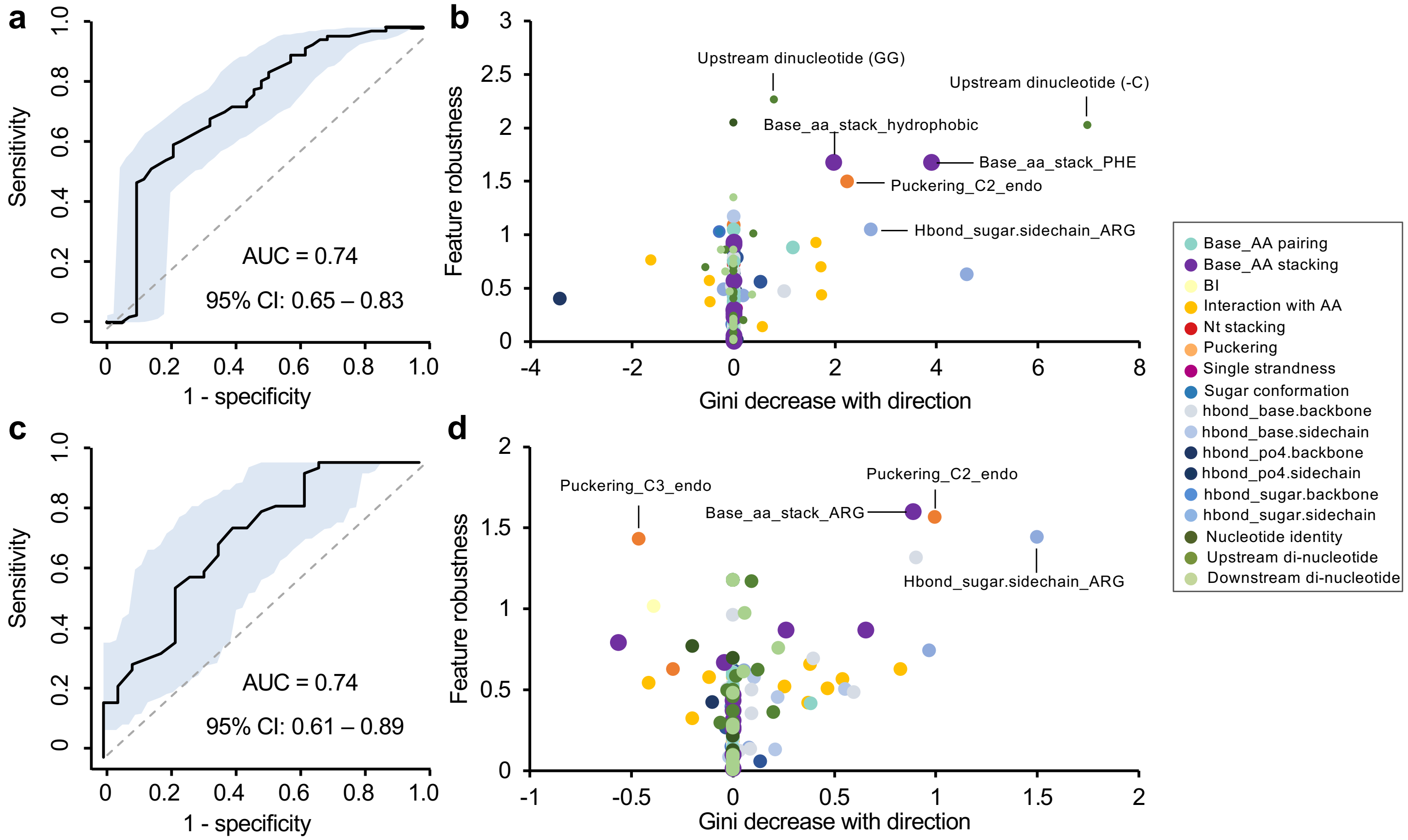

Supplementary Fig. 5: Prediction of crosslinked vs. non-crosslinked nucleotides using random forest trained with nucleotide and overlapping di-nucleotide identities together with structural features. a, Prediction performance of crosslinked vs. non-crosslinked nucleotides as measured by AUC (black curve). The shaded area indicates 95% confidence interval as determined by 2000 models trained with bootstrapped data. **b**, Feature importance plot with Mean GiniDecrease of each feature shown in x-axis and feature robustness derived from permutation tests shown in y-axis. The direction of Mean GiniDecrease represents whether the feature is positively or negatively associated with crosslinking. Different feature groups are color-coded. **c,d,** Similar to (a,b) but the analysis is limited to crosslinked vs. non-crosslinked nucleotides stacking with aromatic amino acids.

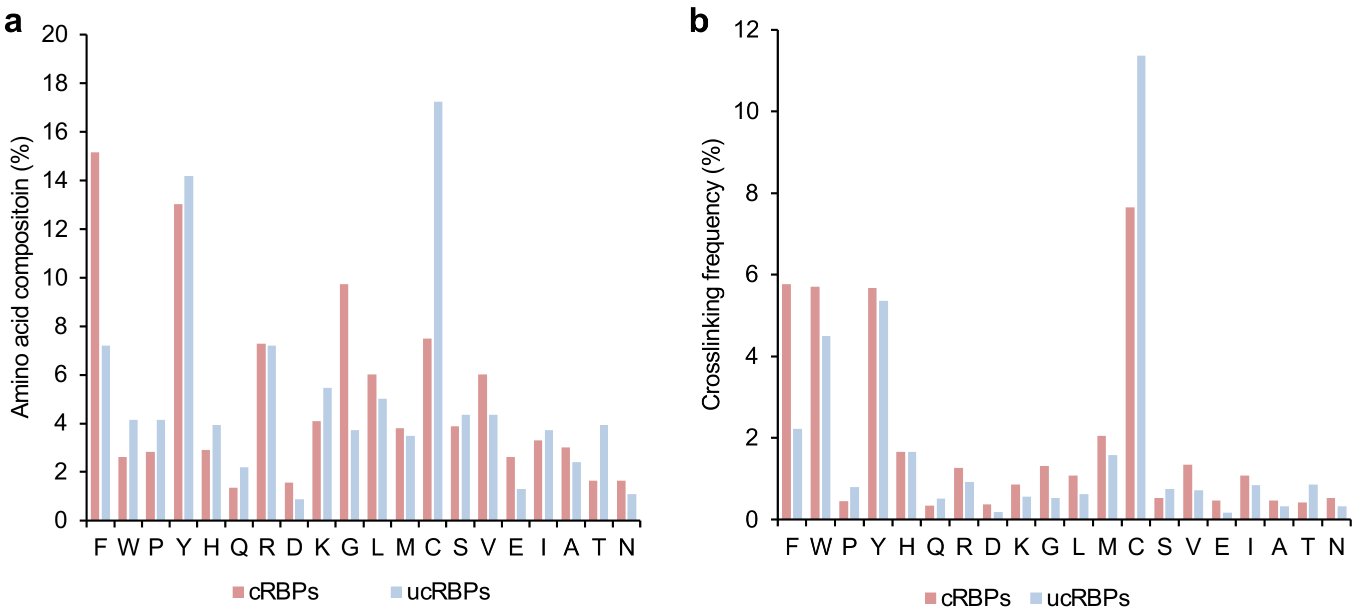

Supplementary Fig. 6: Comparison of amino acid crosslinking between conventional and unconventional RBPs. **a**, Amino acid composition for conventional (cRBPs) and unconventional RBPs (ucRBPs). **b**, Amino acid crosslinking frequencies estimated separately for cRBPs and ucRBPs. For both panels, amino acids are ranked as in Fig. 4b in the main text.

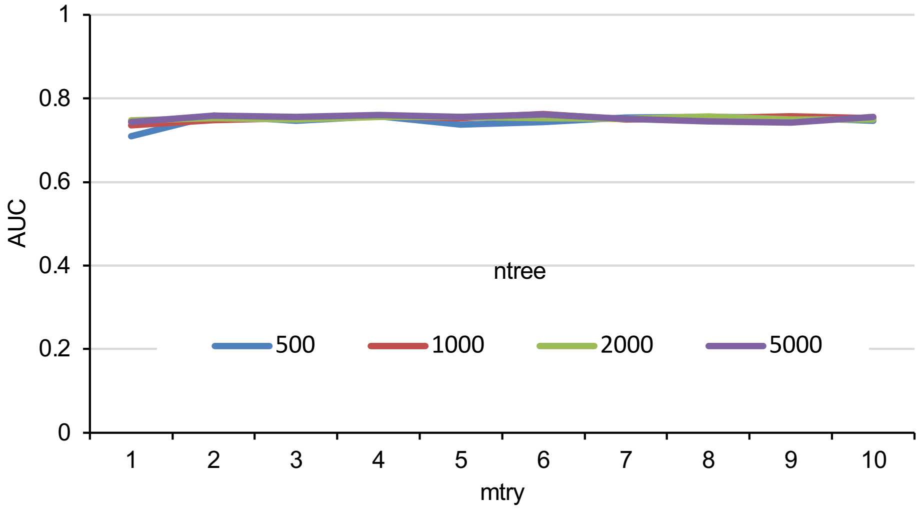

Supplementary Fig. 7: Prediction performance of crosslinked vs. non-crosslinked amino acids using random forest models trained with different model parameters. A wide range of model parameters including the number of trees (ntree) in the forest and the number of features per tree (mtry) are tested, and the AUC of each model is plotted.

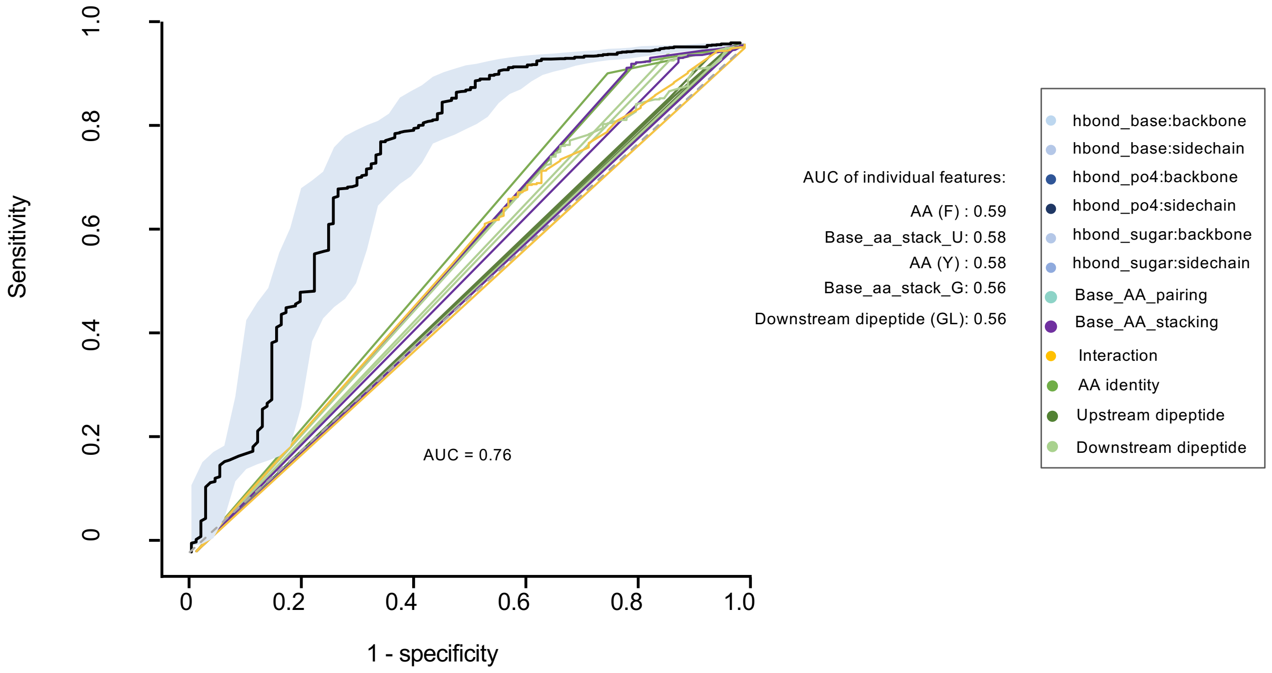

Supplementary Fig. 8: Prediction performance of crosslinked vs. non-crosslinked amino acids using all features combined or individual features. The ROC of the random forest model using all features is shown by the black curve. The shaded area indicates 95% confidence interval as determined by 2000 models trained with bootstrapped data. The ROCs of individual features are also shown, with color codes indicated on the right. The AUCs of the top individual features are also indicated.

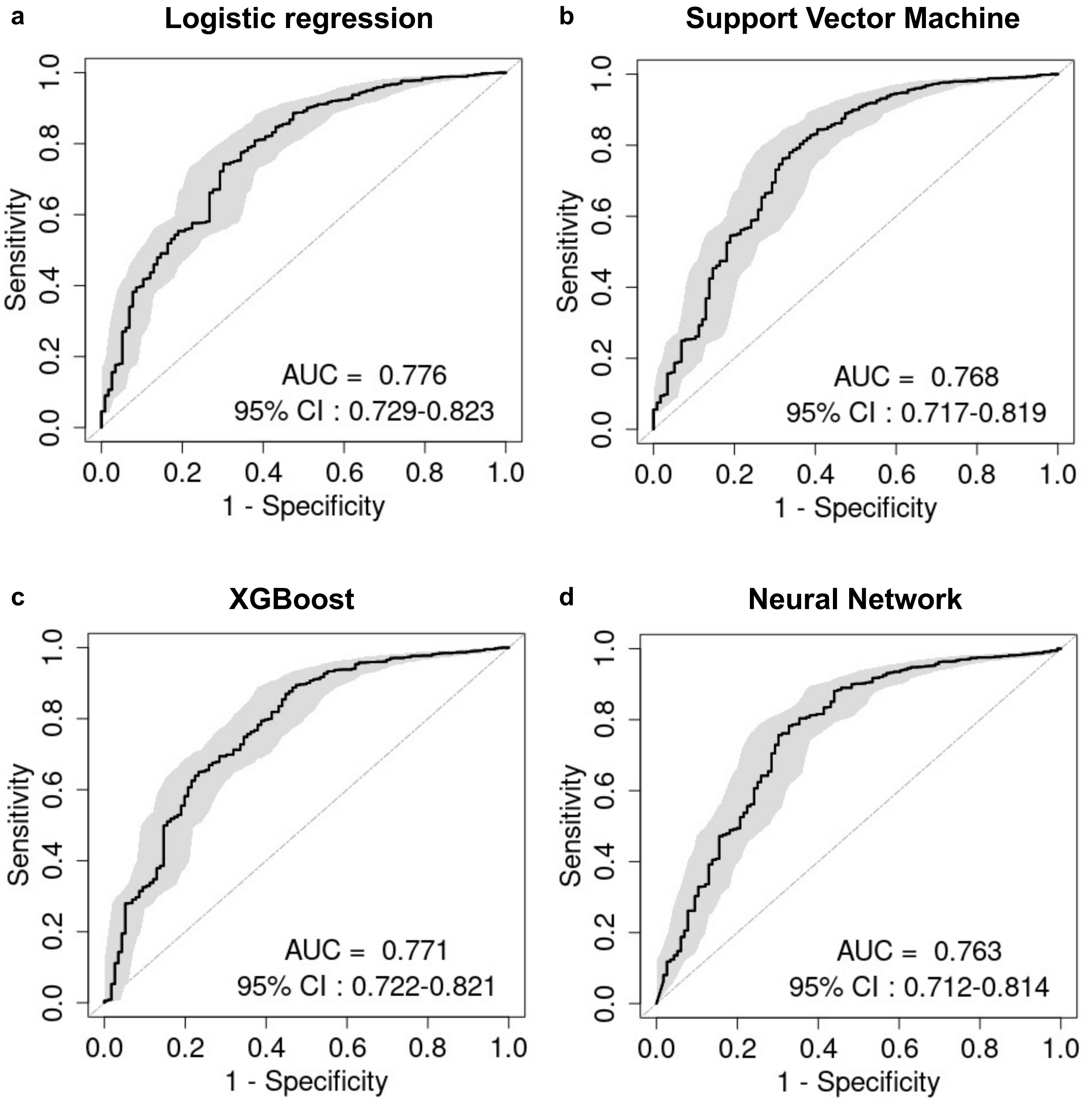

Supplementary Fig. 9: Prediction performance of crosslinked vs. non-crosslinked amino acids using different classification methods. a, Logistic regression. **b**, Support vector machine (SVM). **c**, XGBoost. **d**, Neural network. In each panel, the prediction performance of crosslinked vs. non-crosslinked amino acids as measured by ROC AUC (black curve). The shaded area indicates the 95% confidence interval as determined by 2000 models trained with bootstrapped samples.

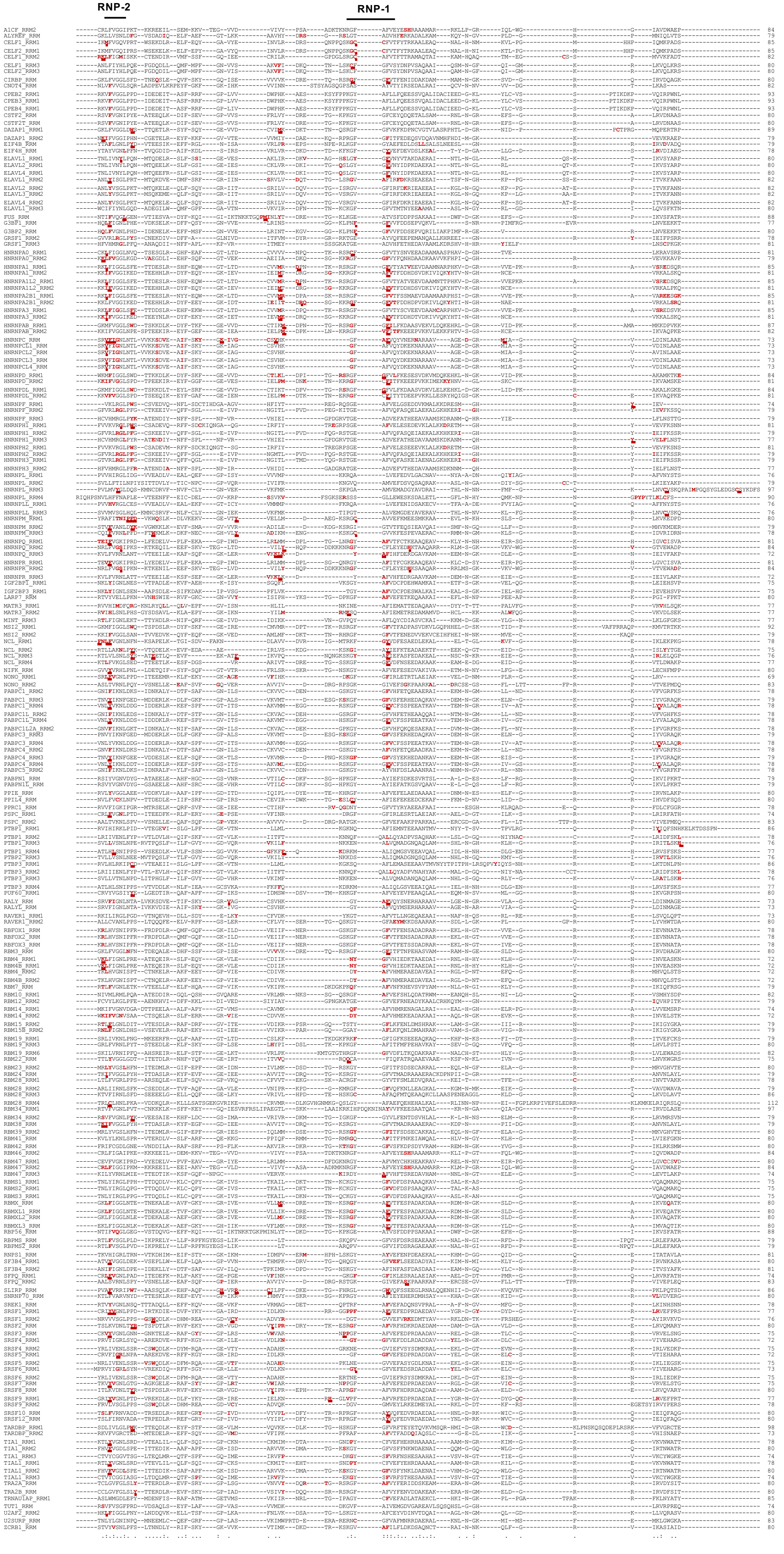

RNP-2
RNP-1

Supplementary Fig. 10. Crosslinked amino acids in RRMs. Multiple sequence alignments of RRMs are shown with crosslinked amino acids (data from Bae et al.) highlighted in red. Underscored amino acids have ≥10 spectrum counts.

```
                               GXXG
QKI_KH        YVPVKEYPDFNFVGRILGPRGLTAKQLEAETGC-KIMVRGKG---------------------------SMRDKKKEEQ--NRGKPNWEHLNEDLHV-----------------------
SF1_KH        MIPQDEYPEINFVGLLIGPRGNTLKNIEKECNA-KIMIRGKG---------------------------SVKEGKVGRKDGQ----MLPGEDEPLHALVTANTMEN-VKKAVEQI-----
KHDRBS1_KH    ----------NFVGKILGPQGNTIKRLQEETGA-KISV----------------------------------------------------------------------------------
KHDRBS2_KH    LIPVKQYPKFNFVGKLLGPRGNSLKRLQEETGA-KMSILGKG---------------------------SMRDKAKEEELRKSGEAKYAHLSDELHVLI---------------------
KHDRBS3_KH    LIPVKQFPKFNFVGKLLGPRGNSLKRLQEETLT-KMSILGKG---------------------------SMRDKAKEEELRKSGEAKYFHLNDDL-------------------------

NOVA1_KH3     KDVVEIAVPENLVGAILGKGGKTLVEYQELTGA-RIQISKKG---------------------------EFVPGTRNRKVTITGTPAATQAAQYLI------------------------
NOVA2_KH3     KELVEIAVPENLVGAILGKGGKTLVEYQELTGA-RIQISKKG---------------------------EFLPGTRNRRVTITGSPAATQAAQYLI------------------------
NOVA1_KH2     ANQVKIIVPNSTAGLIIGKGGATVKAVMEQSGA-WVQLSQKPD----------------------------GINLQERVVTVSGEPEQNRKAVELI------------------------
NOVA2_KH2     AKQAKLIVPNSTAGLIIGKGGATVKAVMEQSGA-WVQLSQKPE----------------------------GINLQERVVTVSGEPEQVHKAVSAI------------------------
NOVA1_KH1     QYFLKVLIPSYAAGSIIGKGGQTIVQLQKETGA-TIKLSKLSKSK------------------------DFYPGTTERVCLIQGTVEALNA---VHGFI---------------------
NOVA2_KH1     EYFLKVLIPSYAAGSIIGKGGQTIVQLQKETGA-TIKL---SKSK------------------------DFYPGTTERVCLVQGTAEALNA---VHSFI---------------------

PCBP1_KH1     TLTIRLLMHGKEVGSIIGKKGESVKRIREESGA-RINISEGN--------------------------------CPERIITLTGPTNAIFKAFAMI------------------------
PCBP2_KH1     TLTIRLLMHGKEVGSIIGKKGESVKKMREESGA-RINISEGN--------------------------------CPERIITLAGPTNAIFKAFAMI------------------------
PCBP3_KH1     TLTIRLLMHGKEVGSIIGKKGETVKKMREESGA-RINISEGN--------------------------------CPERIVTITG------------------------------------
PCBP4_KH1     TLTLRMLMHGKEVGSIIGKKGETVKRIREQSSA-RITISEGS--------------------------------CPERITTITG------------------------------------
PCBP1_KH3     QTTHELTIPNNLIGCIIGRQGANINEIR-QMSGAQIKIANPV------------------------------EGSSGRQVTITGSAASISLAQYLI------------------------
PCBP2_KH3     TTSHELTIPNDLIGCIIGRQGAKINEIR-QMSGAQIKIANPV------------------------------EGSTDRQVTITGSAASISLAQYLI------------------------
PCBP3_KH3     ASTHELTIPNDLIGCIIGRQGTKINEIR-QMSGAQIKIANAT------------------------------EGSSERQITITGTPANISLAQYLI------------------------
PCBP4_KH3     TSSQEFLVPNDLIGCVIGRQGSKISEIR-QMSGAHIKIGNQA------------------------------EGAGERHVTITG------------------------------------
PCBP1_KH2     PVTLRLVVPATQCGSLIGKGGCKIKEIRESTGA-QVQVAGDM-----------------------------LPNSTERAITIAGVPQSVTECVKQI------------------------
PCBP2_KH2     PVTLRLVVPASQCGSLIGKGGCKIKEIRESTGA-QVQVAGDM-----------------------------LPNSTERAITIAGIPQSIIECVKQI------------------------
PCBP3_KH2     PVTLRLVVPASQCGSLIGKGGSKIKEIRESTGA-QVQVAGDM-----------------------------LPNSTERAVTISG------------------------------------
PCBP4_KH2     PVTLRLVIPASQCGSLIGKAGTKIKEIRETTGA-QVQVAGDL-----------------------------LPNSTERAVTVSG------------------------------------
HNRNPK_KH2    DCELRLLIHQSLAGGIIGVKGAKIKELRENTQT-TIKLFQEC-----------------------------CPHSTDRVVLIGGKPDRVVECIKII------------------------
HNRNPK_KH3    IITTQVTIPKDLAGSIIGKGGQRIKQIR-HESGASIKIDEPL------------------------------EGSEDRIITITGTQDQIQNAQYLL------------------------
HNRNPK_KH1    MVELRILLQSKNAGAVIGKGGKNIKALRTDYNA-SVSVPDSS--------------------------------GPERILSISADIETIGEILKKI------------------------

IGF2BP1_KH3   QEMVQVFIPAQAVGAIIGKKGQHIKQLSRFASA-SIKIAPPE-----------------------------TPDSKVRMVIITGPPEAQFKAQGRI------------------------
IGF2BP3_KH3   TETVHLFIPALSVGAIIGKQGQHIKQLSRFAGA-SIKIAPAE-----------------------------APDAKVRMVIITGPPEAQFKAQGRI------------------------
IGF2BP2_KH3   QEIVNLFIPTQAVGAIIGKKGAHIKQLARFAGA-SIKIAPAE-----------------------------GPDVSERMVIITGPPEAQFKAQGRI------------------------
IGF2BP1_KH4   KLETHIRVPASAAGRVIGKGGKTVNELQNLTAA-EVVVPRDQT----------------------------PDENDQVIVKIIGHFYASQMAQRKI------------------------
IGF2BP3_KH4   KLEAHIRVPSFAAGRVIGKGGKTVNELQNLSSA-EVVVPRDQT----------------------------PDENDQVVVKITGHFYACQVAQRKI------------------------
IGF2BP2_KH4   KLEAHIRVPSSTAGRVIGKGGKTVNELQNLTSA-EVIVPRDQT----------------------------PDENEEVIVRIIGHFFASQTAQRKI------------------------
IGF2BP1_KH1   DIPLRLLVPTQYVGAIIGKEGATIRNITKQTQS-KIDVHRKE-----------------------------NAGAAEKAISVHSTPEGCSSACKMI------------------------
IGF2BP3_KH1   DLPLRLLVPTQFVGAIIGKEGATIRNITKQTQS-KIDVHRKE-----------------------------NAGAAEKSITILSTPEGTSAACKSI------------------------
IGF2BP2_KH1   DFPLRILVPTQFVGAIIGKEGLTIKNITKQTQS-RVDIHRKE-----------------------------NSGAAEKPVTIHATPEGTSEACRMI------------------------
IGF2BP1_KH2   EVPLKILAHNNFVGRLIGKEGRNLKKVEQDTET-KITISSLQ---------------------------DLTLYNPERTITVKGAIENCCRAEQEI------------------------
IGF2BP3_KH2   EIPLKILAHNNFVGRLIGKEGRNLKKIEQDTDT-KITISPLQ---------------------------ELTLYNPERTITVKGNVETCAKAEEEI------------------------
IGF2BP2_KH2   EIPLKILAHNGLVGRLIGKEGRNLKKIEHETGT-KITISSLQ---------------------------DLSIYNPERTITVKGTVEACASAEIEI------------------------

FUBP1_KH2     NAVQEIMIPASKAGLVIGKGGETIKQLQERAGV-KMVMIQDG---------------------------PQ-NTGADKPLRITGDPYKVQQAKEMV------------------------
KHSRP_KH2     GTVQEIMIPAGKAGLVIGKGGETIKQLQERAGV-KMILIQDG---------------------------SQ-NTNVDKPLRIIGDPYKVQQACEMV------------------------
FUBP3_KH2     STIQEILIPASKVGLVIGRGGETIKQLQERTGV-KMVMIQDG---------------------------PL-PTGADKPLRITGDAFKVQQAREMV------------------------
FUBP1_KH1     VMTEEYKVPDGMVGFIIGRGGEQISRIQQESGC-KIQIAPDS------------------------------GGLPERSCMLTGTPESVQSAKRLL------------------------
KHSRP_KH1     SMTEEYRVPDGMVGLIIGRGGEQINKIQQDSGC-KVQISPDS------------------------------GGLPERSVSLTGAPESVQKAKMML------------------------
FUBP3_KH1     VITEEFKVPDKMVGFIIGRGGEQISRIQAESGC-KIQIASES------------------------------SGIPERPCVLTGTPESIEQAKRLL------------------------
FUBP1_KH3     NEGIDVPIPRFAVGIVIGRNGEMIKKIQNDAGV-RIQFKPDD------------------------------GTTPERIAQITGPPDRCQHAAEII------------------------
KHSRP_KH3     GGGIDVPVPRHSVGVVIGRSGEMIKKIQNDAGV-RIQFKQDD------------------------------GTGPEKIAHIMGPPDRCEHAARII------------------------
FUBP3_KH3     GGSIEVSVPRFAVGIVIGRNGEMIKKIQNDAGV-RIQFKPDD------------------------------GISPERAAQVMGPPDRCQHAAHII------------------------
FUBP1_KH4     LQEFNFIVPTGKTGLIIGKGGETIKSISQQSGA-RIELQRNP---------------------------PPNADPNMKLFTIRGTPQQIDYARQLI------------------------
KHSRP_KH4     GGEMTFSIPTHKCGLVIGRGGENVKAINQQTGA-FVEISRQL---------------------------PPNGDPNFKLFIIRGSPQQIDHAKQLI------------------------
FUBP3_KH4     VQEITYTVPADKCGLVIGKGGENIKSINQQSGA-HVELQRNP---------------------------PPNSDPNLRRFTIRGVPQQIEVARQLI------------------------

FMR1_KH1      ----QFIVREDLMGLAIGTHGANIQQAR-KVPGVT-------------------------------------------------------------------------------------
FXR1_KH1      ----EFVVREDLMGLAIGTHGSNIQQAR-KVPGVT-------------------------------------------------------------------------------------
FXR2_KH1      ----EFTVREDLMGLAIGTHGANIQQAR-KVPGVT-------------------------------------------------------------------------------------
FMR1_KH2      ----VIQVPRNLVGKVIGKNGKLIQEIVDKSGV-V-------------------------------------------------------------------------------------
FXR1_KH2      ----FIQVPRNLVGKVIGKNGKVIQEIVDKSGV-V-------------------------------------------------------------------------------------
FXR2_KH2      ----SVQVPRNLVGKVIGKNGKVIQEIVDKSGV-V-------------------------------------------------------------------------------------

ANKRD17_KH    RRSKKVSVPSTVISRVIGRGGCNINAIR-EFTGAHIDIDKQK------------------------------DKTGDRIITIRGGTESTRQATQLI------------------------

MEX3A_KH1     TTECVPVPTSEHVAEIVGRQGCKIKALRAKTNT-YIKTPVRG---------------------------------EEPVFMVTGRREDVATARREI------------------------
MEX3D_KH1     MTECVPVPSSEHVAEIVGRQGCKIKALRAKTNT-YIKTPVRG---------------------------------EEPVFIVTGRKEDVEMAKREI------------------------
MEX3B_KH1     MTECVPVPSSEHVAEIVGRQGCKIKALRAKTNT-YIKTPVRG---------------------------------EEPVFVVTGRKEDVAMARREI------------------------
MEX3C_KH1     TTECVPVPSSEHVAEIVGRQGCKIKALRAKTNT-YIKTPVRG---------------------------------EEPIFVVTGRKEDVAMAKREI------------------------
MEX3A_KH2     QVTIRVRVPYRVVGLVVGPKGATIKRIQQQTNT-YIITPSRD---------------------------------RDPVFEITGAPGNVERAREEI------------------------
MEX3D_KH2     QTTIQVRVPYRVVGLVVGPKGATIKRIQQRTHT-YIVTPGRD---------------------------------KEPVFAVTGMPENVDRAREEI------------------------
MEX3B_KH2     QTTIQVRVPYRVVGLVVGPKGATIKRIQQQTHT-YIVTPSRD---------------------------------KEPVFEVTGMPENVDRAREEI------------------------
MEX3C_KH2     QTTVQVRVPYRVVGLVVGPKGATIKRIQQQTHT-YIVTPSRD---------------------------------KEPVFEVTGMPENVDRAREEI------------------------

HDLBP_KH4     --------PSWLHRFIIGKKGQNLAKITQQMPKVHIEFTE-----------------------------------GEDKITLEGPTEDVNVAQEQIEGMVKDLINRMDYVEINI------
HDLBP_KH11    --------PQKFHRSVMGPKGSRIQQITRDFSV-QIKFPDREENAVHSTEPVVQENGDEAGEGREAKDCDPGSPRRCDIIIISGRKEKCEAAKEALEALVPVTIEV------EV------
HDLBP_KH5     --------DHKFHRHLIGKSGANINRIKDQYKV-SVRIPPDS--------------------------------EKSNLIRIEGDPQGVQQAKRELLELASRMENE-RTKDLII------
HDLBP_KH9     --------KPEYHKFLIGKGGGKIRKVRDSTGA-RVIFPAAED-------------------------------KDQDLITIIGKEDAVREAQKELEALIQNLDNV-VEDSMLV------
HDLBP_KH2     --------EKAFHPFIAGPYNRLVGEIMQETGT-RINIPPPS--------------------------------VNRTEIVFTGEKEQLAQAVARIKKIYEEKKKKTTTIAVEV------
HDLBP_KH3     --------KKSQHKYVIGPKGNSLQEILERTGV-SVEIPPSD--------------------------------SISETVILRGEPEKLGQALTEVYAKANSFTVSSVAA----------
HDLBP_KH1     --------PKEHHRFVIGKNGEKLQDLELKTAT-KIQIPRPD--------------------------------DPSNQIKITGTKEGIEKARHEVLLISAEQDKRAVE-RLEV------
HDLBP_KH14    --------DHRVHARIIGARGKAIRKIMDEFKV-DIRFPQSGA-------------------------------PDPNCVTVTGLPENVEEAIDHILNLEEEYLADVVDSE-ALQV----
HDLBP_KH12    --------PFDLHRYVIGQKGSGIRKMMDEFEV-NIHVPAPE--------------------------------LQSDIIAITGLAANLDRAKAGLLERVKELQAE--QEDRALRSFKLS
HDLBP_KH6     --------EQRFHRTIIGQKGERIREIRDKFPEVIINFPDPA--------------------------------QKSDIVQLRGPKNEVEKCTKYMQKMVADLVENSYSISVPI------
HDLBP_KH10    --------DPKHHRHFVIRRGQVLREIAEEYGGVMVSFPRSG--------------------------------TQSDKVTLKGAKDCVEAAKKRIQEIIEDLEAQ-VTLECAI------
HDLBP_KH13    --------DPKYHPKIIGRKGAVITQIRLEHDV-NIQFPDKD-----------------------------DGNQPQDQITITGYEKNTEAARDAILRIVGELEQM-VSEDVPL------
HDLBP_KH8     --------PAKLHNSLIGTKGRLIRSIMEECGGVHIHFPVEG--------------------------------SGSDTVVIRGPSSDVEKAKKQLLHLAEEKQTK--SFTVDIRA----
HDLBP_KH7     --------FKQFHKNIIGKGGANIKKIREESNT-KIDLPAEN--------------------------------SNSETIIITGKRANCEAARSRILSIQKDLANI-AEVEVSI------
```

Supplementary Fig. 11: Crosslinked amino acids in KH domains. Multiple sequence alignments of RRMs are shown with crosslinked amino acids (data from Bae et al.) highlighted in red. Underscored amino acids have ≥10 spectrum counts.

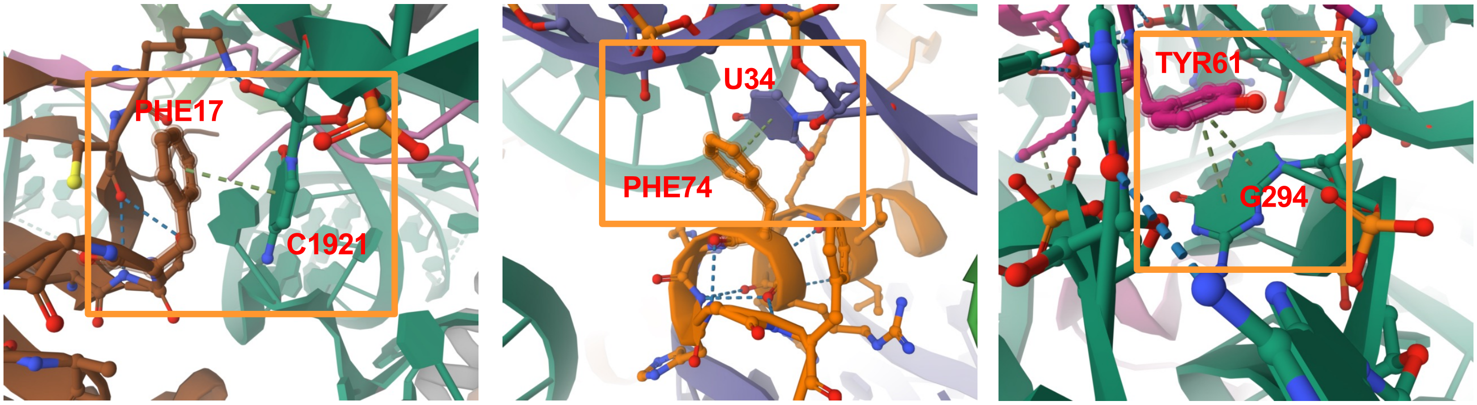

RL14: PHE17-C1921

RL37: PHE74-U34

RL27A: TYR61-G294

Supplementary Fig. 12: Examples of crosslinked aromatic residues directly contacting RNA through base stacking in 80S ribosome. For each example, the crosslinked amino acid and its interacting nucleotide are highlighted.

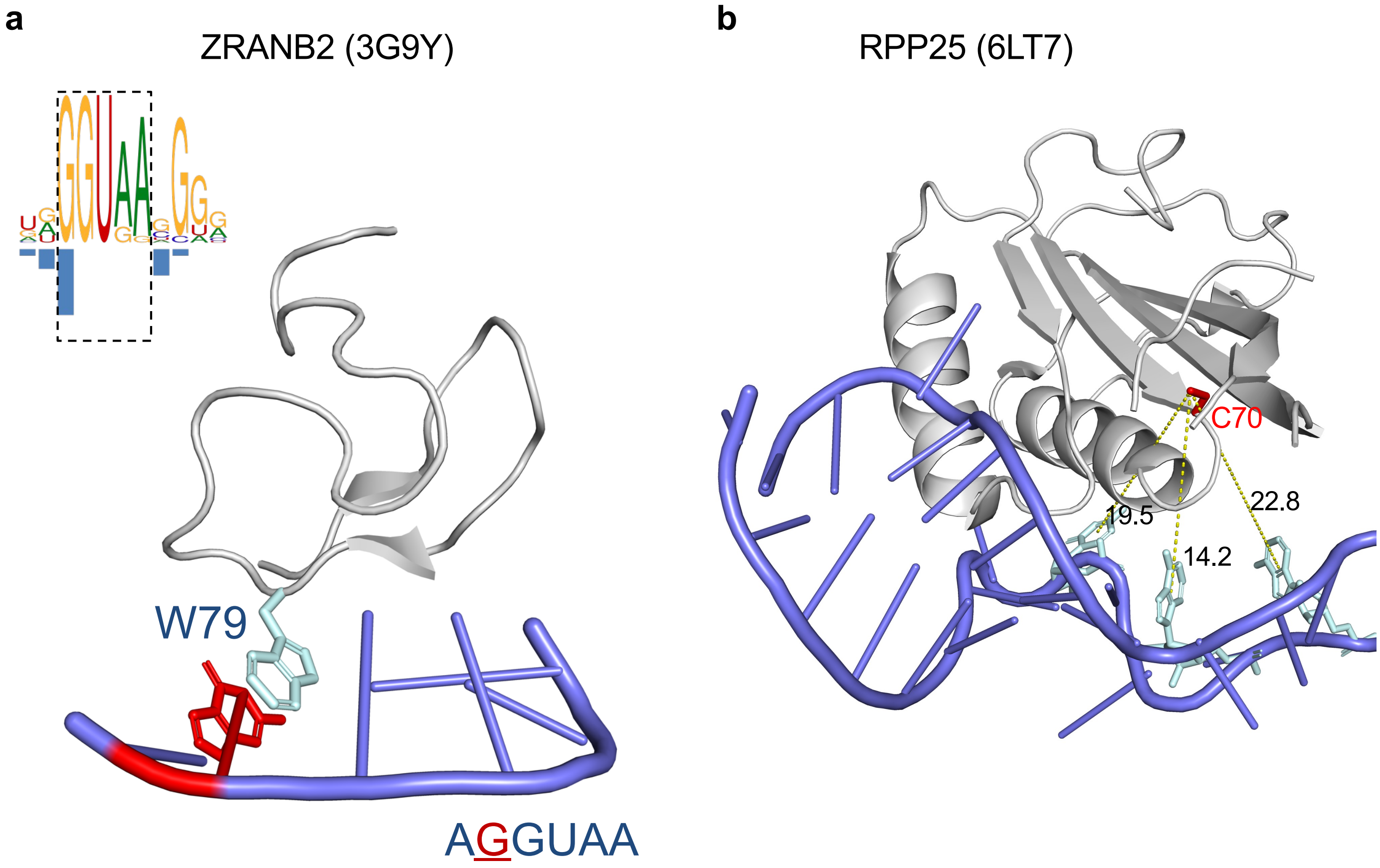

Supplementary Fig. 13. a, ZRANB2 ZNF2 in complex with AGGUAA (PDB accession: 3G9Y). The structure of the protein-RNA complex is illustrated using PyMOL, with RNA in pale blue and protein in gray cartoons. The crosslinked nucleotides (red) in RNA and the crosslinked amino acids (cyan) in protein are shown in sticks to highlight the nucleotide-amino acid contacts. The ZRANB2 RNA-binding sequence motif, as discovered by mCross, is also shown. The bar plot indicates the crosslinking frequencies at different motif positions. **b**, RPP25 in complex with the P3 domain of lncRNA RMRP (PDB accession: 6LT7). The color scheme is as in panel (a). The crosslinked amino acid C70 is highlighted in red. Its distances to the three closest nucleotides are also indicated.
