# Supplementary material for: Structure-based prediction and characterization of photo-crosslinking in native protein–RNA complexes
Source: Nat Commun. 2024 Mar 13;15:2279. doi: 10.1038/s41467-024-46429-y (PMC10937933; doi:10.1038/s41467-024-46429-y)
Supplement: Supplementary file 3 — Description of Additional Supplementary Information [file 41467_2024_46429_MOESM3_ESM.docx]

**Description of additional supplementary files**

**File name: Supplementary Data 1**

**Description: Structural features associated with each nucleotide in protein-RNA complexes**

**File name: Supplementary Data 2**

**Description: Structural features associated with each amino acid in protein-RNA complexes**

**File name: Supplementary Data 3**

**Description: PDB structures inlcuded in the predicton of crosslinked nucleotides**

**File name: Supplementary Data 4**

**Description: RNA nucleotides with asscoiated structural features and prediction results of crosslinking status**

**File name: Supplementary Data 5**

**Description: Feature rankings for prediction of crosslinked nucleotides**

**File name: Supplementary Data 6**

**Description: PDB structures inlcuded for the predicton of crosslinked amino acids**

**File name: Supplementary Data 7**

**Description: Amino acids with asscoiated structural features and the prediction results**

**File name: Supplementary Data 8**

**Description: Feature rankings of prediction of crosslinked amino acids**

**File name: Supplementary Data 9**

**Description: Crosslinked amino acids in the 80s ribosome.** Only amino acids in the structure (PDB: 6Z6M) are included.
