# Supplementary material for: Structure-based prediction and characterization of photo-crosslinking in native protein–RNA complexes
Source: Nat Commun. 2024 Mar 13;15:2279. doi: 10.1038/s41467-024-46429-y (PMC10937933; doi:10.1038/s41467-024-46429-y)
Supplement: Supplementary file 13 — Reporting Summary [file 41467_2024_46429_MOESM13_ESM.pdf]

nature portfolio

Corresponding author(s): Chaolin Zhang

Last updated by author(s): Feb 3, 2024

Reporting Summary

Nature Portfolio wishes to improve the reproducibility of the work that we publish. This form provides structure for consistency and transparency in reporting. For further information on Nature Portfolio policies, see our Editorial Policies and the Editorial Policy Checklist.

Statistics

For all statistical analyses, confirm that the following items are present in the figure legend, table legend, main text, or Methods section.

n/a	Confirmed	
☐	☒	The exact sample size (*n*) for each experimental group/condition, given as a discrete number and unit of measurement
☐	☒	A statement on whether measurements were taken from distinct samples or whether the same sample was measured repeatedly
☐	☒	The statistical test(s) used AND whether they are one- or two-sided<br>*Only common tests should be described solely by name; describe more complex techniques in the Methods section.*
☐	☒	A description of all covariates tested
☐	☒	A description of any assumptions or corrections, such as tests of normality and adjustment for multiple comparisons
☐	☒	A full description of the statistical parameters including central tendency (e.g. means) or other basic estimates (e.g. regression coefficient) AND variation (e.g. standard deviation) or associated estimates of uncertainty (e.g. confidence intervals)
☐	☒	For null hypothesis testing, the test statistic (e.g. *F*, *t*, *r*) with confidence intervals, effect sizes, degrees of freedom and *P* value noted<br>*Give P values as exact values whenever suitable.*
☒	☐	For Bayesian analysis, information on the choice of priors and Markov chain Monte Carlo settings
☒	☐	For hierarchical and complex designs, identification of the appropriate level for tests and full reporting of outcomes
☒	☐	Estimates of effect sizes (e.g. Cohen's *d*, Pearson's *r*), indicating how they were calculated

Our web collection on statistics for biologists contains articles on many of the points above.

Software and code

Policy information about availability of computer code

Data collection	No commercial, open source, or custom software or code was used for data collection.
Data analysis	CTK (v1.1.3)<br>mCross (v1.0.0)<br>3DNA (v2.3)<br>R (v3.6.2)<br>Caret (v6.0-80)<br>ggplot2 (v3.3.5)<br>pROC (v1.16.2)<br>Custom codes link: https://github.com/chaolinzhanglab/PxR3D

For manuscripts utilizing custom algorithms or software that are central to the research but not yet described in published literature, software must be made available to editors and reviewers. We strongly encourage code deposition in a community repository (e.g. GitHub). See the Nature Portfolio guidelines for submitting code & software for further information.

Data

Policy information about availability of data

All manuscripts must include a data availability statement. This statement should provide the following information, where applicable:

- Accession codes, unique identifiers, or web links for publicly available datasets
- A description of any restrictions on data availability
- For clinical datasets or third party data, please ensure that the statement adheres to our policy

Protein-RNA complex structures were downloaded from Protein Data Bank (PDB; https://www.rcsb.org). The PDB accession codes of protein-RNA complex structures analyzed in this study were included in Supplementary Data 3 and 6. The CLIP data analyzed in this study were downloaded from NCBI Sequence Read Archive (https://www.ncbi.nlm.nih.gov/sra) or the ENCODE website (https://www.encodeproject.org) with accession codes summarized in Supplementary Data 3.

Research involving human participants, their data, or biological material

Policy information about studies with human participants or human data. See also policy information about sex, gender (identity/presentation), and sexual orientation and race, ethnicity and racism.

Reporting on sex and gender	n/a
Reporting on race, ethnicity, or other socially relevant groupings	n/a
Population characteristics	n/a
Recruitment	n/a
Ethics oversight	n/a

Note that full information on the approval of the study protocol must also be provided in the manuscript.

Field-specific reporting

Please select the one below that is the best fit for your research. If you are not sure, read the appropriate sections before making your selection.

☒ Life sciences ☐ Behavioural & social sciences ☐ Ecological, evolutionary & environmental sciences

For a reference copy of the document with all sections, see nature.com/documents/nr-reporting-summary-flat.pdf

Life sciences study design

All studies must disclose on these points even when the disclosure is negative.

Sample size	Sample size was determined by the availability of data.
Data exclusions	In each protein-RNA complex strcuture, we excluded the protein and RNA chains that are redundant. Amino acid and nucleotide residues without direct contacts were also excluded.
Replication	We verified the structural features contributing to crosslinking by prediction of crosslinked nucleotides and crosslinked amino acids. The prediction accuracy was evaluated using 10 fold cross validation, and the robustness of the results was also evaluated using a range of model parameters as well as different machine-learning based classifieres.
Randomization	The samples were divided into ten folds randomly to evaluate prediction accuracy by cross-validation.
Blinding	The was no blinding in the datasets we used.The samples in the study consist of individual nucleotides or amino acids, which are inherently objective and unbiased in nature. Given that the samples themselves do not possess any inherent variation or potential for bias, the application of blinding is not applicable in this context. Blinding is typically employed in studies where there is a need to conceal treatment or intervention information from participants or researchers to minimize subjective biases. However, since the focus of this study is on individual molecular units, blinding does not play a role in ensuring objectivity or reducing bias in the prediction process.

Reporting for specific materials, systems and methods

We require information from authors about some types of materials, experimental systems and methods used in many studies. Here, indicate whether each material, system or method listed is relevant to your study. If you are not sure if a list item applies to your research, read the appropriate section before selecting a response.

Materials & experimental systems

n/a	Involved in the study
☒	☐ Antibodies
☒	☐ Eukaryotic cell lines
☒	☐ Palaeontology and archaeology
☒	☐ Animals and other organisms
☒	☐ Clinical data
☒	☐ Dual use research of concern
☒	☐ Plants

Methods

n/a	Involved in the study
☒	☐ ChIP-seq
☒	☐ Flow cytometry
☒	☐ MRI-based neuroimaging
